# Forecasting the Long-Term Trends of Coronavirus Disease 2019 (COVID-19) Epidemic Using the Susceptible-Infectious-Recovered (SIR) Model

**DOI:** 10.3390/idr13030063

**Published:** 2021-07-29

**Authors:** Agus Kartono, Savira Vita Karimah, Setyanto Tri Wahyudi, Ardian Arif Setiawan, Irmansyah Sofian

**Affiliations:** Department of Physics, Faculty of Mathematical and Natural Sciences, IPB University (Bogor Agricultural University), Jalan Meranti, Building Wing S, 2nd Floor, Kampus IPB Dramaga, Bogor 16680, Indonesia; saviravitak@gmail.com (S.V.K.); stwahyudi@apps.ipb.ac.id (S.T.W.); aarif@apps.ipb.ac.id (A.A.S.); irmansyah@apps.ipb.ac.id (I.S.)

**Keywords:** compartment, COVID-19, epidemic, estimation, pandemic

## Abstract

A simple model for predicting Coronavirus Disease 2019 (COVID-19) epidemic is presented in this study. The prediction model is presented based on the classic Susceptible-Infectious-Recovered (SIR) model, which has been widely used to describe the epidemic time evolution of infectious diseases. The original version of the Kermack and McKendrick model is used in this study. This included the daily rates of infection spread by infected individuals when these individuals interact with a susceptible population, which is denoted by the parameter *β*, while the recovery rates to determine the number of recovered individuals is expressed by the parameter *γ*. The parameters estimation of the three-compartment SIR model is determined through using a mathematical sequential reduction process from the logistic growth model equation. As the parameters are the basic characteristics of epidemic time evolution, the model is always tested and applied to the latest actual data of confirmed COVID-19 cases. It seems that this simple model is still reliable enough to describe the dynamics of the COVID-19 epidemic, not only qualitatively but also quantitatively with a high degree of correlation between actual data and prediction results. Therefore, it is possible to apply this model to predict cases of COVID-19 in several countries. In addition, the parameter characteristics of the classic SIR model can provide information on how these parameters reflect the efforts by each country to prevent the spread of the COVID-19 outbreak. This is clearly seen from the changes of the parameters shown by the classic SIR model.

## 1. Introduction

In mid-March 2020, the World Health Organization (WHO) declared that the 2019 Coronavirus Disease (COVID-19) was a global pandemic, caused by severe acute respiratory syndrome virus SARS-CoV-2 [1]. Because the COVID-19 pandemic has spread globally, this pandemic creates a global threat to public health. As a result of this pandemic, most countries closed their borders to prevent the spread of COVID-19 widely, causing global economic growth to decrease as a result of this pandemic. The closure of borders by governments in some countries is carried out to reduce the infection rate and mortality, and also minimize the impact of the inevitable decline in economic growth [2].

Patients who are infected by COVID-19 with mild symptoms, will still experience moderate to severe symptoms. Although the effect may be mild in patients with mild symptoms, they will still experience fever, cough, spasms, myalgia, and fatigue. On the other hand, patients with moderate and severe symptoms of the virus can experience acute respiratory distress syndrome (ARDS) and severe pneumonia, which may result in multi-organ failure in these patients [3,4]. In most patients with severe symptoms, SARS-CoV-2 is very interesting to observe, as it can complicate inflammatory cytokines, marked by increased interleukin 6 (IL-6). Several recent clinical trials of COVID-19 have shown that IL-6 levels are higher in the group of patients with severe symptoms than in the group of patients with moderate symptoms [5,6].

The measures to control the COVID-19 pandemic involve a very complex process for several reasons. Firstly, no medicine can cure the disease caused by this new virus [7]. Secondly, the origin of the virus that causes this disease is still unclear, although several hypotheses have been suggested that the virus originated in bats [8]. This hypothesis requires further detailed research to confirm this hypothesis. Thirdly, the incubation period of this disease is based on clinical studies in patients, and has been confirmed to be about 14 days; however, in most cases, symptoms only appear after 4–5 days after the patient is infected with the virus [9]. During the incubation period, an asymptomatic patient may not know that they are infected, meaning that the patient can transmit the virus to other individuals without being aware. A recent study has found that this virus can remain on the surface of an object for 9 days [10], thus adding to the complexity of avoiding the spread of this virus. Other studies have also concluded that the rate of infection of the disease is very fast, resulting in a significant burden on health facilities hosting a number of infected patients [11].

The situation of a pandemic will increase the demand for epidemiological mathematical models [12], in order to explain the dynamics of the outbreak. This not only provides an explanation, but also has the potential to predict when an outbreak is new and is still in an active phase. This prediction aims to quickly estimate the impact of the COVID-19 pandemic on a population in the future. This is to ensure that the spread prevention measures that will be taken by the public health system are faster and more effective, such as tracing and quarantine for individuals who have come into contact with infected individuals.

A large number of epidemiological models have been proposed during the COVID-19 pandemic. A variety of models have been developed, ranging from very simple models to complex models that include many variables and parameters. However, complex models can include many parameters and variables, hence, the process of estimating these parameters requires detailed statistics and validation. Factors affecting the statistics of these parameters and variables should have real data available [13,14,15,16]. This may not be available in every country, as there may be very limited data available. Therefore, support for the application of a simple model in the form of a compartment regression, which is commonly referred to as the Susceptible-Infectious-Recovered (SIR) and Susceptible-Exposed-Infectious-Recovered (SEIR) models, is still very relevant [17,18,19,20,21]. These epidemiological models are in the form of ordinary differential equations and contain several parameters and variables; furthermore, these parameters have information that is immediately available to be applied in measures in order to prevent the spread of an outbreak.

The analytical solution to the SIR epidemic model has been obtained accurately. The solution uses an asymptotic approach, and conforms to the old asymptotic behavior of the epidemic model. The analytical solution was applied to the COVID-19 epidemic [22]. An effective approach has been demonstrated by the SIR epidemic model to describe the current COVID-19 epidemic. This model has been applied to populations in China, South Korea, India, Australia, Italy, and the US. The SIR model can predict the spread of COVID-19 if there is a control scenario in the community, by considering appropriate restrictions and strong policies to be implemented to control the infection rate from an early stage of the spread of COVID-19 [23]. The standard SIR compartment model and a modified version have been proposed to account for the percentage of infected cases, with the assumption that homogeneous population mixing in the SIR model is still conventional. This model is applied to predict outcomes in India using certain interventions, with the predicted *R*_0_ value for India still being greater than 1.00 [24]. A new forecasting-based method has been proposed using the SIRD model, meaning that there is a modification of the SIR model with the addition of a death compartment (*D*). The parameters of the modified model were estimated by using machine learning to better suit the actual data. The model has been applied to the spread of COVID-19 in São Paulo, Brazil, and the USA [25,26].

This study aims to predict the parameters of the classic SIR model to evaluate the severity impact of the COVID-19 epidemic over the short and long term, and also estimate the curve of the cumulative number of COVID-19 infected cases in several countries. This model is the earliest and simplest epidemiological model, and has been proposed by Kermack and McKendrick [27]. Their model approach is one of the most popular basic epidemiological mathematical models [28]. This model divides the population into three compartments, Susceptible (*S*), Infectious (*I*), and Recovered (*R*), which always interact kinetically. This study also uses the logistic growth model regression to estimate the SIR model parameters for several countries for the short and long term. This regression method is a newly applied method to generate predictions of *R*_0_ parameters through the SIR model that can be used to plan and prepare health systems in these countries.

Section 2 describes the classic SIR model, starting with introducing the equations of the SIR model and then the method for deriving two parameters of the model that plays an important role in understanding the characteristics of an epidemic in an area. Section 3 describes the logistic growth regression method for estimating the two parameters of the SIR model. Section 4 describes the numerical methods. Section 5 deals with the prediction results and discussion of the SIR model. The last section, Section 6, contains the conclusions of this study.

## 2. The Classic Susceptible-Infected-Recovered (SIR) Model

The basic reproductive rate, *R*_0_, also known as the baseline reproduction ratio, is the average number of secondary infections that occur when the primary infectious agent is introduced into a highly vulnerable population. Based on the above definition, it is implicitly assumed that an infected individual will remain in the population for the entire period of the infection process. Therefore, the individual will mix with other individuals in the population in the same condition. In epidemiological models, the basic reproduction rate, *R*_0_, is often used as a threshold measure to determine the severity of an infectious disease in a population.

The classic SIR model is composed of a system of equations, consisting of the three coupled ordinary differential equations (ODEs). This simple model can be applied easily to gain insight into how the COVID-19 virus spreads in a population as a function of time, and it can also predict the probability of an increase in susceptible individuals in the population. The classic SIR model was designed to remove many of the complexities associated with the spread of viruses as a real-time evolution. This classic SIR model is also useful for predicting the spread of the virus, both qualitatively and quantitatively. The classic SIR model is a dynamic system, consisting of three coupled ODEs which describe the time evolution of three populations as follows:

1. The compartment of a susceptible individual, *S*(*t*), represents the number of individuals who have not been infected, but these individuals can be infected. Susceptible individuals can become infected or remain in a susceptible condition when the virus spreads from its source or a new source occurs. The more individuals that are infected, the more susceptible the population; therefore, it will increase the susceptible population over a period of time.
2. The compartment of an individual infected by the virus, *I*(*t*), is an individual who is already infected, and this individual can transmit to susceptible individuals. These individuals who are infected will remain infected, but these individuals can be excluded from the population if these infected individuals recover or die.
3. The compartment of the recovered individual, *R*(*t*), is the individual who has recovered from the virus, and these individuals are assumed to have immunity to the virus; therefore, these individuals also have herd immunity.

The classic SIR model has several important assumptions, namely:

(1) The total population does not change much over time, this means that *dS*(*t*)/*dt* + *dI*(*t*)/*dt* + *dR*(*t*) = 0.
(2) Every individual in a population has the same health characteristics. It is characterized by these individuals having the same immunity, hence, their immune response to the virus is the same.
(3) In the population, each individual interacts with one another.
(4) Any infected individual can transmit and spread the disease in susceptible populations.

The classic SIR model describes an infectious disease that spreads in a population with permanent immunity. If an infectious disease spreads in a population in a relatively short time span of less than one year, the disease outbreak is usually called an epidemic. Therefore, this model does not include the birth variable. One of these infectious diseases is caused by a virus. The infectious diseases caused by viruses, such as Middle East Respiratory Syndrome Coronavirus (MERS-CoV), varicella-zoster virus (VZV), and COVID-19, can be classified as epidemics. The epidemiological model in compartment form for the SIR model can be defined as follows (Equations (1)–(3)):

(1) $$\frac{dS\left(t\right)}{dt}=-\frac{\beta }{N}I\left(t\right)S\left(t\right)\;,\;\;\;\;S\left(0\right)=S_{0}\geq 0$$

(2) $$\frac{dI\left(t\right)}{dt}=\frac{\beta }{N}I\left(t\right)S\left(t\right)-\gamma I\left(t\right)\;,\;\;\;\;I\left(0\right)=I_{0}\geq 0$$

(3) $$\frac{dR\left(t\right)}{dt}=\gamma I\left(t\right)\;,\;\;\;\;R\left(0\right)=R_{0}\geq 0$$

where *N* (*N* = *S*(*t*) + *I*(*t*) + *R*(*t*)) defines the total number of individuals in the population. In this model, the basic reproductive rate, *R*_0_, remains the same over time because neither susceptibility nor infection levels have occurred after spread; therefore, the threshold quantity of the basic reproductive rate is given by $R_{0}=\frac{\beta }{\gamma }$.

The rate of infected individuals is determined by the interaction between the infected individuals and the susceptible individuals, and this is related to the constant *β*. This means that the level of spread of infectious disease occurs when the infected individuals interact daily with the susceptible populations. Meanwhile, the rate of individual recovery is determined by the rate of recovery constant, which is related to the constant *γ*.

As the dynamics of the COVID-19 epidemic are much faster than births, demographic factors can be ignored. The classic SIR model is a simple epidemiological model in the form of compartments, which is often used for modelling infectious diseases. The population number is defined as *N* >> 1 individual, which is divided into three compartments, namely: *S* (susceptible), *I* (infected), and *R* (recovered). Each individual in the population can fit between these three compartments. In constant population numbers, the variables *S*(*t*), *I*(*t*), and *R*(*t*) can determine the susceptible, infected, and recovered fractions of individuals in the population who are involved in the infection process at time *t*, where (Equation (4)):

(4) S(t)+I(t)+R(t)=1 ,

Because the variables, *S*(*t*), *I*(*t*), and *R*(*t*) are fractions, the variables will always be in the interval (0, 1). If the parameters *β*(*t*) and *γ*(*t*) define the infection process rate and the recovery rate is time-dependent and is always positive, then the classic SIR model can be defined by two dynamic equations, namely Equations (1) and (2). If Equation (2) is substituted into Equation (1), then Equation (1) can be written as follows (Equation (5)):

(5) $$\frac{dS\left(t\right)/dt}{S\left(t\right)}=\frac{d}{dt}\left(\ln S\left(t\right)\right)=-\beta \left(t\right)I\left(t\right)\;,$$

where (Equation (6))

(6) $$I\left(t\right)=-\frac{1}{\beta \left(t\right)}\frac{d}{dt}\left(\ln S\left(t\right)\right)\;.$$

Equation (3) is the dynamic equation of *R*(*t*), which follows Equation (4) for the limit of addition is (Equation (7)):

(7) $$\frac{dR\left(t\right)}{dt}=-\frac{d}{dt}\left[S\left(t\right)+I\left(t\right)-1\right]=\gamma \left(t\right)I\left(t\right)\;,$$

So that Equation (4) can be as follows (Equation (8)):

(8) $$\frac{dR\left(t\right)}{dt}=-\frac{d}{dt}\left[S\left(t\right)+I\left(t\right)-1\right]=\gamma \left(t\right)I\left(t\right)\;,\;\frac{dR\left(t\right)}{dt}=-k\left(t\right)\frac{d}{dt}\left(\ln S\left(t\right)\right).$$

So that the inverse of the time-dependent reproductive factor can be expressed as follows (Equation (9)):

(9) $$k\left(t\right)=\frac{\gamma \left(t\right)}{\beta \left(t\right)}\;.$$

The analytical solution approach that is derived in this work will hold for any *β*(*t*), as long as *γ*(*t*) and *β*(*t*) remain proportional to each other [29]. If *k* is a constant, then it is usually denoted as an inverse of the basic reproduction number, $k=\frac{1}{R_{0}}.$

In the qualitative case over time, the ratio k=γ(t)/β(t) can be assumed to be an identical constant over time, as long as *t* = −∞ to *t* = ∞. This implies that the boundary conditions must be used, so that *S*(−∞) = 1, *I*(−∞) = 0, and *R*(−∞) = 0 because the epidemic cannot exist at time *t* = −∞. In a special case, the ratio value *k* = 0 and *γ*(*t*) = 0, where *R*(*t*) = 0 for each time is based on Equation (8), whereas Equations (1), (2) and (4) can be reduced to I(t)=1−S(t), in order to form a simple logistic differential equation dS(t)/dt=−β(t)S(t)[1−S(t)] which can determine the parameter *β*(*t*). If the variable is equal to the constant (*I*(*t*) = *I*_0_), then the following equation is obtained (Equation (10)):

(10) $$\frac{dR\left(t\right)}{dt}=\gamma I_{0}\;$$

Then, Equation (9) is integrated, so that the following equation is obtained (Equation (11)):

(11) $$R\left(t\right)=\gamma tI_{0}\;,$$

If it is necessary to recover during time *t* = *T*, then the variable $R\left(T\right)=I_{0}$ or γT=1, and the following equation is obtained (Equation (12)):

(12) $$\gamma \approx \frac{1}{T}\;,$$

where *T* defines the recovery period. If the change in time is known to be dt=a, then from Equation (3) the following equation is obtained (Equation (13)):

(13) $$\frac{R\left(t+a\right)}{a}=\gamma I$$

or (Equation (14))

(14) $$\gamma \approx \frac{R\left(t+1\right)-R\left(t\right)}{I\left(t\right)}\;.$$

The actual data is used to directly estimate, to obtain the parameter estimated of *γ*.

## 3. The Logistic Growth Regression

The logistic growth model is a regression model that has been widely applied in epidemiological mathematical models to estimate the growth rate and the reduction in the cumulative infected cases [30]. This model assumes that the exponential growth of the cumulative infected cases at the start of the epidemic is followed by a steadily increasing growth, and is ended by a decreasing growth rate. Based on the literature [31], the logistic growth model is derived from the population growth model in the field of ecology; then, in 1838, the Malthus population model was improved by Pierre François Verhulst and defined as follows (Equation (15)):

(15) $$\frac{dC\left(t\right)}{dt}=rC\left(t\right)\left(1-\frac{C\left(t\right)}{K}\right).$$

**Figure 1:**
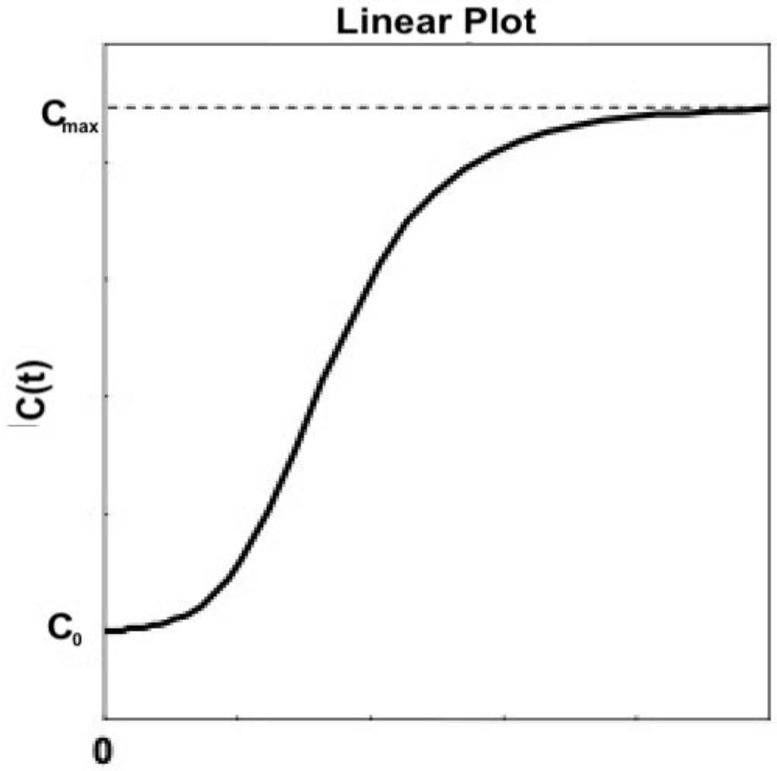
An example of a simulation result of a logistic growth *S*-shaped curve plotted on linear coordinate, using Equation (15) as a model.

The variable *C*(*t*) defines the number of infected cases at a given time *t*, while the constant *r* defines the infected rate in the population, and the constant *K* defines the final size of infected cases at the time of the end of the epidemic. The variable *C*(*t*) is calculated from the logistic growth equation over time and will produce an *S*-shaped curve (as seen in Figure 1), while dC(t)/dt defines population growth. Therefore, at the initial time *t* = 0, it is known that the initial condition is *C*(0) = *C*_0_. The number of infected cases is defined as follows (Equation (16)):

(16) $$C\left(t\right)=\frac{K}{1+A\;\exp\left(-r\left(t\right)\right)}\;,$$

and the constant *A* is defined as follows (Equation (17)):

(17) $$A=\frac{K-C_{0}}{C_{0}}\;.\;$$

The initial boundary is the number of infected cases at *t* = 0, that is, C(0)=K/(1+A). While the turning point is $t_{p}=ln\left(A/r\right)$, the curve condition of the cumulative infected cases changes when there is a rapid increase in the number of the cumulative infected cases; then, it changes to a rapid decrease in the number of the cumulative infected cases. The simulation will initialize *A*, *r*, and *K* randomly and update them using the non-linear least-squares method [32]. The boundary condition for the parameter *r* is set according to the epidemic situation in each time period, then the COVID-19 growth model with the logistic growth model equation will estimate the parameters *A*, *r*, and *K*. The parameters calculated from the logistic growth model will be applied to predict the growth pattern of COVID-19.

Equations (16) and (17) are logistic growth functions that follow the sigmoid curve and can be stated as follows (Equation (18)):

(18) $$C\left(t\right)=\frac{KC_{0}e^{rt}}{K+C_{0}\left(e^{rt}-1\right)}\;.$$

This study did not use a logistic growth function to directly estimate the cumulative infected cases. However, this logistic function was used to fit the SIR deterministic model to predict the cumulative infected cases, define the deterministic model parameters, and fit the deterministic model to the actual data. The first derivative of the logistic growth function will be applied to predict newly infected cases each day. Therefore, Equation (18) substituted for Equation (15) will obtain the logistic differential equation as follows (Equation (19)):

(19) $$\frac{dC\left(t\right)}{dt}=f\left(t,\alpha \right)=\frac{rKC_{0}e^{rt}\left(K-C_{0}\right)}{{\left(K+C_{0}e^{rt}-C_{0}\right)}^{2}}$$

The function f(t,α) with parameters $\alpha =\left(K,C_{0},r\right)$ defines the number of new cases observable each day with an error *ε*, so that in a statistical model, the equation can be expressed as *I*(*t*) (Equation (20)):

(20) I(t)=f(t,α)+ε(t) ,     where t=1, 2, …, T.

Equation (20) is the key equation for the time-series data model used in the non-linear least-squares method to estimate the parameters and to minimize the residual (error) sum of squares (Equation (21)):

(21) $$w\left(t\right)=\overset{T}{\underset{t=1}{\sum }}{\left(I\left(t\right)-f\left(t,\alpha \right)\right)}^{2}$$

Von Bertalanffy growth models (VBGM) can also be applied in this study because the distribution of growth samples usually deviate from normality. This is due to asymmetry, in which the curve will produce tails. The application of non-linear regression analysis of the VBGM can also consider additive random error, meaning that the VBGM is more flexible to being modeled asymmetrically [33].

The parameters *α* = (*K*, *C*_0_, *r*) are estimated as a predictive value at a certain time *t* using the total sum of squares ($TSS=\overset{T}{\underset{i=1}{\sum }}{\left(I\left(t\right)\right)}^{2}$), regression sum of squares ($RSS=\overset{T}{\underset{i=1}{\sum }}{\left(f\left(t,\alpha \right)\right)}^{2}$), and the residual (error) sum of squares ($ESS=\overset{T}{\underset{i=1}{\sum }}\;w{\left(t\right)}^{2}$). Furthermore, according to the statistical identity that *TSS* = *RSS* + *ESS*, the predicted results of parameters *α* need to be adjusted to the actual data, using the coefficient of determination *R*^2^ which is defined as follows (Equation (22)):

(22) $$R^{2}=\frac{RSS}{TSS}=\frac{\sum_{i=1}^{T}{\left(f\left(t,\alpha \right)\right)}^{2}}{\sum_{i=1}^{T}{\left(I\left(t\right)\right)}^{2}}\;.$$

Equation (22) will be used for input data and also for estimating parameters using the logistic growth model. The regression of the logistic model will adjust to the test data, based on its *R*^2^ value. The *R*^2^ value will identify that when the predicted value approaches 0 within a certain period for the test data, new cases remains sporadic and the *R*^2^ value becomes unstable. However, the *R*^2^ value close to 1 indicates that the new case findings will be consistent with the actual data. The *R*^2^ value may differ among parts of the non-linear curve; therefore, logistic regression is used to compare the fit between the sections.

## 4. Numerical Solutions

Several popular numerical methods for solving coupled ordinary differential equations (ODEs) have varying speed and accuracy, such as the Euler, Runge–Kutta, Adams–Bashforth, and Adams–Moulton methods. This study proposes that the implementation of the Runge–Kutta numerical method be used to solve the equations of the classic SIR model. This is suggested as the Runge–Kutta method is a numerical method that offers a good balance between efficiency and accuracy for the epidemiological mathematical model. The Equations (1)–(3) are solved based on the periods of actual data collection in the logistic growth regression for an infectious compartment *I*(*t*) only (Equations (20) and (21)), so that each parameter of the SIR equations will be estimated based on the actual data in each period.

This research simulation consists of two main implementation stages: (i) calculation of SIR model equations and (ii) logistic growth regression process. The algorithm of the simulation includes:

- The numerical equation solver of the SIR model (Equations (1)–(3)) that uses the Runge–Kutta method.
- The logistic growth regression process that uses the calculation results from the infected compartment *I*(*t*), and the actual data from infected cases for each period (Equations (20) and (21)).
- The process of assessing the determinant coefficient (*R*^2^) (Equation (22)) of the curve between the results of the *I*(*t*) calculation and the actual data of infected cases for each period.
- The process of estimating the parameters of the SIR model using Equations (5)–(9).
- If the conditions are met (the *R*^2^ value is more than 90%), then the simulation is stopped and the results are reported; however, if the conditions are not met, return to step III.
- The process of outputting the results to be stored in a file.

In the algorithm above, solving the equations of the SIR model, determining the estimated parameters of the model, and calculating the determinant coefficients are encoded using MATLAB R2018b, and the simulation is carried out on a laptop with an Intel Core i5 Dual-Core 2.9 GHz Processor and 8GB DDR3 1867MHz RAM.

## 5. Results and Discussion

This study will analyze the dynamics spread of COVID-19 using an epidemiological model, namely the classic SIR model, to predict the final total of the cumulative infected cases and the final date of the COVID-19 epidemic. This model will be used to estimate the spread of COVID-19 in Singapore, Saudi Arabia, the Philippines, and Indonesia. The parameters of this model are estimated using actual data from the number of cumulative infected cases reported by the World Health Organization (WHO), starting from early confirmed cases who infected COVID-19 in each country, until the end of December 2020. The actual data above will be applied as input data to estimate the parameters of the SIR model, which will be updated daily by WHO through the online COVID-19 dashboard [34].

The most important parameter for determining the intrinsic transmission of COVID-19 that can be estimated from the classic SIR model is the basic reproduction number *R*_0_. The *R*_0_ value defines the average number of infected individuals that a single infectious agent can cause during the period of infection, without any intervention. In the real scenario, the *R*_0_ value is assumed to be constant; however, in the development of an epidemic, the more interventions that are carried out to control the spread of the epidemic will gradually reduce the *R*_0_ value. In this case, the basic reproductive rate, *R*_0_, can be generalized to a dynamic value. Therefore, it can be defined as the mean number of secondary infected cases caused by infection over time *t*, as shown in Equation (9).

The prediction simulation of the classic SIR model requires the determination of the parameter *R*_0_ value. The regression technique of the logistic growth model is used to help the SIR model adjust to the actual data that has been reported, while the range of the parameter *R*_0_ value needs to be adjusted to suit epidemiological rationality. The most recent actual data of cumulative confirmed cases were obtained from the WHO Coronavirus (COVID-19) Dashboard (https://covid19.who.int/, accessed on 23 December 2020) [34]. The new confirmed cases are collected every day. Based on the consideration of possible different medical resources and interventions in different regions, the classic SIR model will be applied to actual data from four different country regions: (1) Singapore, (2) Saudi Arabia, (3) the Philippines, and (4) Indonesia, to see the difference in the phenomenon.

To estimate the long-term trend of the number of confirmed cases, it is necessary to have actual data on cases reported to the public, starting from the first confirmed cases as a starting point for the input data of this model. This model will use the same actual data to make predictions of the four countries to test the validity of this model.

### 5.1. The COVID-19 Epidemic Case in Singapore

**Figure 2:**
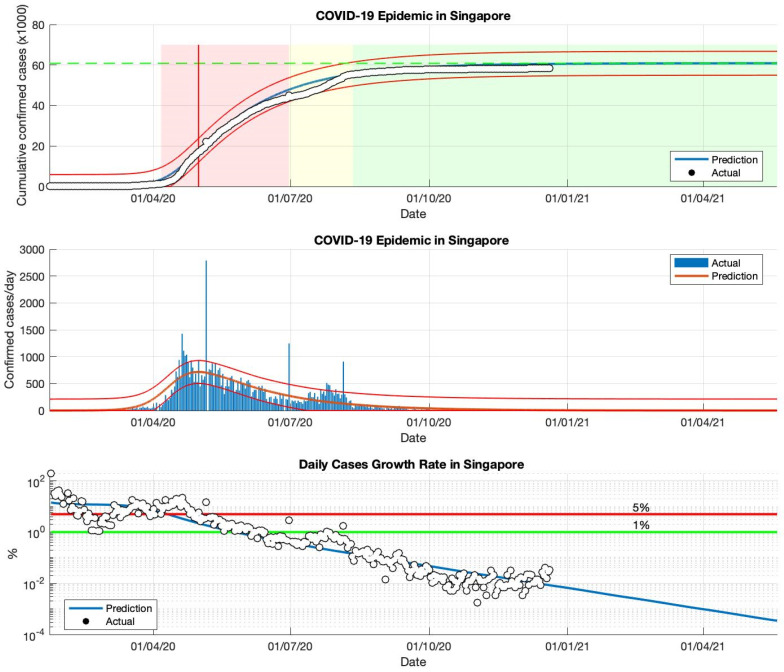
The above figure shows the predicted results of the SIR model for cumulative infected cases, compared with the actual data (the two red lines between the black lines represent a predictive error limit of about 5%, while the green dotted line predicts the total size of infected cases in the final phase of the epidemic). The red line intersects the curve of cumulative infected cases to predict the peak point of the epidemic. This line is parallel to the peak of the curve of the middle figure. The middle figure shows the predicted results of the SIR model for daily infected cases. The below figure shows the daily case growth rate (expressed in %), calculated based on the difference between the total number of infected cases today and the total number of infected cases yesterday, divided by the number of cumulative infected cases. These figures are the prediction results of the epidemic cases in Singapore, in the period from 23 January to 23 December 2020.

The prediction results of the number of cumulative COVID-19 cases in Singapore have been estimated and are presented in Figure 2. The epidemic curve can be said that the cumulative cases of COVID-19 in Singapore have decreased at a rate from the end of September 2020 to the end of December 2020. In addition, it can be seen that the cumulative cases have reached around 58.550 as of 22 May 2020, with the average daily confirmed case rates below 1% from mid-September 2020 to the end of December 2020. The COVID-19 cases in Singapore are expected to end at the end of December 2020, with cumulative cases of around 59.786 based on the WHO database [34]. According to this study, the daily new cases in Singapore decreased starting from the beginning of October. The daily new cases in Singapore reported by the World Health Organization (WHO) averaged below 100 after the end of December 2020. This means that the COVID-19 epidemic is under control in Singapore. This also means that the COVID-19 epidemic in Singapore can be estimated well using the classic SIR model as a model of prediction or estimation of the spread of COVID-19.

In Table 1, the prediction results use actual data from daily infected cases in the first and second (short-term) periods in Singapore, to estimate the parameters of the SIR model. The estimation results of these parameters indicate that the epidemic process is ongoing because the *R*_0_ value is still greater than 1.00. Figure 2 shows some of the peaks in the time series of new daily COVID-19 cases in Singapore. The proposed SIR model uses data from the first period, 23 January to 23 May 2020, but it still does not predict the peak of the epidemic in the period. Furthermore, this model uses more actual data as input data for the estimation process by extending the time limit to estimate the epidemic prediction results, set to 23 June and 23 September 2020, respectively. Then, simulation in this study is continued by using more actual data as input data for the parameter estimation process of *R*_0_. The actual data of daily infected cases has been extended to 23 December 2020 (long-term) to estimate the predicted results of the epidemic. It can be seen from the results of the *R*_0_ value, which is close to 1.00, although it is not yet equal to 1.00. This means that daily infected cases in Singapore have started to decline. It is observed that this model has shown better adaptation to the actual data. This model provides a good estimate of the epidemic at an early stage. Although there is only one case of maximum increase (peak) or several maximum increases (peaks), the fitting between the actual data and the prediction results is always based on the analysis of the *R*^2^ value. This study has also shown that each period shows a decrease in the daily growth rate of COVID-19 infected cases (see Figure 2 and Table 1), based on the analysis performed by the parameter *R*_0_. This study suspects that it is due to the better prevention of virus transmission by the government. It is also supported by the community’s behavior to implement better health protocols (wear a mask, keep distancing, wash hands using antiseptic). The correlation between the prediction results and the actual data based on the *R*^2^ value also shows that there is a good correlation for each period.

### 5.2. The COVID-19 Epidemic Case in Saudi Arabia

**Figure 3:**
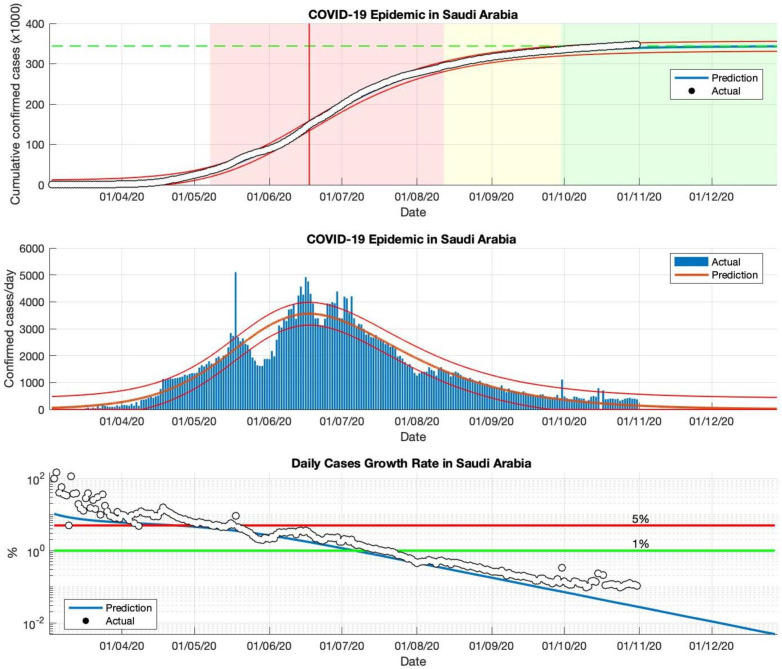
The above figure shows the predicted results of the SIR model for cumulative infected cases, compared with the actual data (the two red lines between the black lines represent a predictive error limit of about 5%, while the green dotted line predicts the total size of infected cases in the final phase of the epidemic). The red line intersects the curve of cumulative infected cases to predict the peak point of the epidemic. This line is parallel to the peak of the curve of the middle figure. The middle figure shows the predicted results of the SIR model for daily infected cases. The below figure shows the daily case growth rate (expressed in %) calculated based on the difference between the total number of infected cases today and the total number of infected cases yesterday, divided by the number of cumulative infected cases. These figures are the prediction results of the epidemic case in Saudi Arabia in the period from 2 March to 23 December 2020.

The SIR model has also been used to predict the cumulative virus-infected cases in Saudi Arabia, from the start of the epidemic on 2 March to 23 December 2020. The prediction results are shown in Figure 3 and Table 2. The first and second periods are actual data from daily infected cases in Saudi Arabia, which are used as input data to estimate the parameters of the SIR model. The results of this simulation predict short-term estimates of the epidemic in Saudi Arabia; the parameter estimation results are presented in Table 2. Based on the parameter estimation results, it can be seen that the *R*_0_ value is still above 1.00. This means that the epidemic is ongoing and the epidemic is still having an impact in Saudi Arabia. This is also reinforced by the daily growth rate of infected cases, which remains above 1%.

Furthermore, long-term forecasting (third and fourth periods) of the actual data of daily infected cases in Saudi Arabia is presented in Table 2. The cumulative prediction results of COVID-19 infected cases in Saudi Arabia are arranged in four periods. From 2 March to 23 December 2020, the estimated cumulative number of COVID-19 infected cases in Saudi Arabia is around 361.110, the actual data is around 361.178 based on WHO sources. The prediction results obtained from the SIR model show that the prediction results are close to the actual data. Based on the estimation results of the parameters *R*_0_ (close to the value 1.00), and the daily growth rate of cases infected with COVID-19 (the value has been below 1%) obtained using this model, there is a downward trend in the epidemic in Saudi Arabia. All predicted results are shown in Figure 3 and Table 2. The correlation between the prediction results and the actual data based on the *R*^2^ value also shows that there is a good correlation for each period in Saudi Arabia.

### 5.3. The COVID-19 Epidemic Case in the Philippines

**Figure 4:**
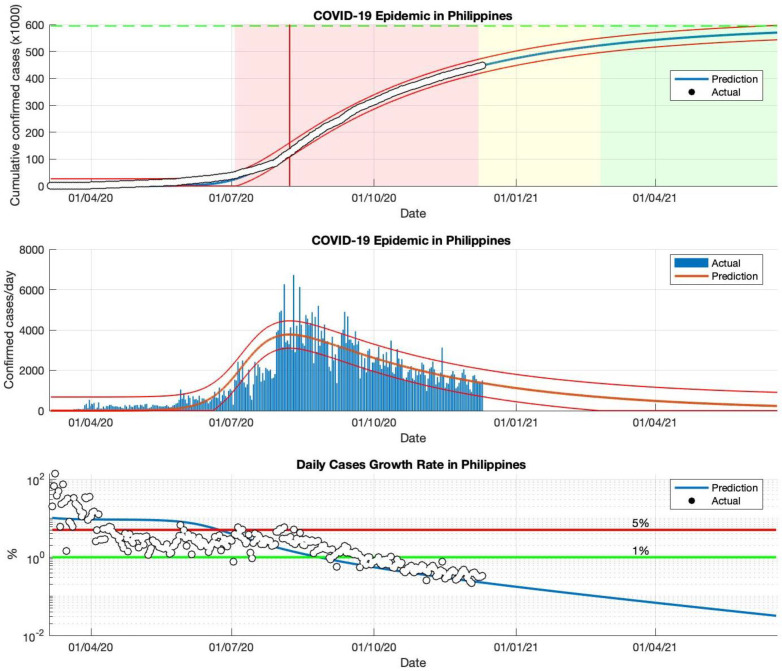
The above figure shows the predicted results of the SIR model for cumulative infected cases, compared with the actual data (the two red lines between the black lines represent a predictive error limit of about 5%, while the green dotted line predicts the total size of infected cases in the final phase of the epidemic). The red line intersects the curve of cumulative infected cases to predict the peak point of the epidemic. This line is parallel to the peak of the curve of the middle figure. The middle figure shows the predicted results of the SIR model for daily infected cases. The below figure shows the daily case growth rate (expressed in %) calculated, based on the difference between the total number of infected cases today and the total number of infected cases yesterday, divided by the number of cumulative infected cases. These figures are the prediction results of the epidemic case in the Philippines in the period from 5 March to 23 December 2020.

The actual data from the COVID-19 infected cases in the Philippines were taken from 23 January to 23 December 2020, then the actual data was divided into four periods. In the first and second periods, the estimation results of the *R*_0_ parameters still show a value greater than 1.00, meaning that the epidemic in the Philippines remains ongoing and has an impact. This is also reinforced by the daily growth rate of infected cases in the Philippines, which is still above 1%. The prediction results are shown in Figure 4 and Table 3.

The simulation is continued by using actual data as estimation data. In the third and fourth periods, the prediction results show that the *R*_0_ parameters have approached 1.00, although not yet equal to 1.00. This indicates that the epidemic in the Philippines has begun to decline. However, the daily growth rate of infected cases has not significantly fallen below 1%.

The prediction results of the SIR model predict that there will be at least 599.784 COVID-19 infected cases by the time the epidemic ends in the Philippines, as shown in Figure 4. The estimated daily growth rate of COVID-19 infected cases in the Philippines is still fluctuating. Table 3 also shows that the value of the parameter *R*_0_ is not close to 1.00, which means that the cumulative number of cases infected with COVID-19 in the Philippines can still double if there is no serious prevention of the spread of the virus from the government.

Based on the epidemic case in the Philippines, the logistic growth regression method shows consistent results and follows actual data, this is based on the *R*^2^ value for each epidemic period. The SIR model identifies that if the prediction results are close to the new daily infected cases that are sporadic or fluctuating, this model remains stable to follow the sporadic or fluctuating pattern. This is based on the *R*^2^ value which remains in the interval of 90–100% and the value is not below 90%.

### 5.4. The COVID-19 Epidemic Case in Indonesia

**Figure 5:**
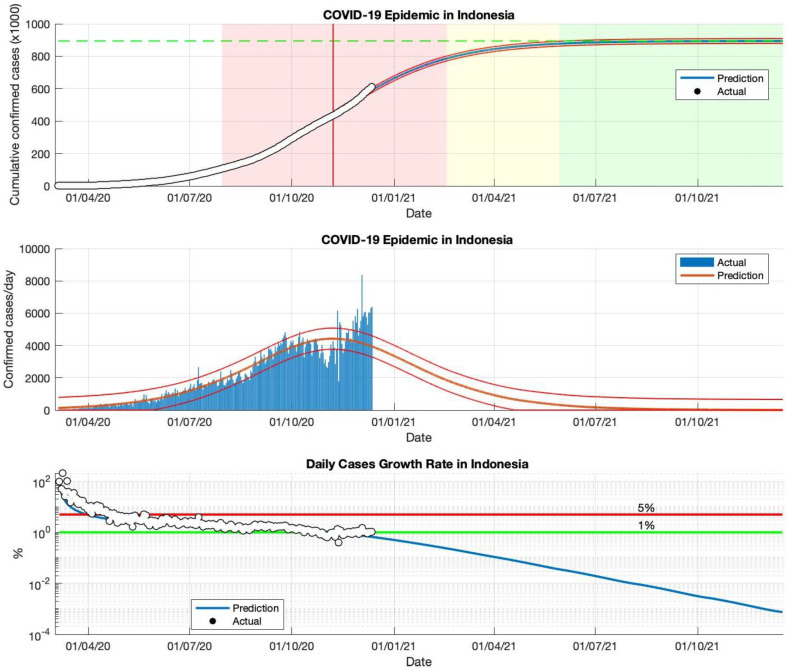
The above figure shows the predicted results of the SIR model for cumulative infected cases compared with the actual data (the two red lines between the black lines represent a predictive error limit of about 5%, while the green dotted line predicts the total size of infected cases in the final phase of the epidemic). The red line intersects the curve of cumulative infected cases to predict the peak point of the epidemic, this line is parallel to the peak of the curve of the middle figure. The middle figure shows the predicted results of the SIR model for daily infected cases. The below figure shows the daily case growth rate (expressed in %), calculated based on the difference between the total number of infected cases today and the total number of infected cases yesterday, divided by the number of cumulative infected cases. These figures are the prediction results of the epidemic case in Indonesia in the period from 2 March to 23 December 2020.

This SIR model has also been applied to actual data from daily COVID-19 infected cases in Indonesia. Epidemic cases in Indonesia remain actively contagious; this is based on actual data from WHO [34], which records that cases of COVID-19 infection continue to increase. The prediction results of the SIR model also show that COVID-19 transmission remains high, and the outbreak is far from over (see Figure 5 and Table 4).

In the case of an epidemic in Indonesia, the period of actual data input is divided into four periods, based on the actual data collected when the first case of COVID-19 appeared, starting 2 March 2020. Then, the actual data was input as an estimation of *R*_0_ parameters until 2 May, 2 July, 2 September, and 23 December 2020. Based on the prediction results of the *R*_0_ parameters, which remains above 1.00, the epidemic in Indonesia is still ongoing or the virus transmission process is still ongoing. This is reinforced by the prediction results of the daily growth rate of infected cases that are still above 1%. The government must do better in handling the transmission of the virus. The screening of individuals in contact with infected individuals needs to be increased. Individuals who have been infected need to be quarantined immediately.

The prediction results of this model result in an estimated cumulative number of infected cases of around 850.504 in the final phase of the COVID-19 pandemic (as shown in Figure 4). The cumulative infected cases will increase, as this model still estimates the daily growth rate of infected cases remains above 1%. Each period has a determinant coefficient value (*R*^2^) above 95%, which indicates that the prediction results follow the actual data.

## 6. Conclusions

This model is presented to offer some advantages of the simple classic SIR model. This model also has certain limitations, including not being able to predict the daily fluctuating infected cases that may arise during the epidemic. This simple type of epidemiological model is limited to only three population compartments (*S*, *I*, *R*), thus, this model is a simple deterministic model. In addition, this model does not consider age, gender, or other factors. This model assumes that there is homogeneous mixing in the population. This means that individuals in the population can make random contact, and that the transmission rate and recovery rate for all individuals are the same. This model also assumes that there is no vaccine is available, the total population size is constant, and that each individual who has recovered will acquire permanent immunity.

The main strength of this model is that it is simple, yet the predictions or estimations can include the large population size. Although this model relies on only two parameters, the results obtained can adjust directly to the actual data reported from the daily population of individuals infected with COVID-19. If the effect of the intervention and preventative action has been implemented for spread of COVID-19, this can be concluded based on parameter values that can adjust to the actual data reported. Therefore, this model will be very useful if these parameters are obtained early in the epidemic because these parameters can be evaluated after the effects of vaccines, a pharmaceutical and medical intervention that has been implemented, or, if there will be the peaks of a second epidemic or multiple waves (sub-epidemic). However, the daily number of infected individuals in the population fluctuates, as in Singapore and the Philippines.

An additional advantage of this model is that the value of the parameters of this model, obtained from data on confirmed COVID-19 cases reported from various countries, can be used to predict the quantity value of *R*_0_. This value is an essential measure of how each country has been affected by the COVID-19 epidemic. The value of *R*_0_ describes a quantitative assessment of the severity of the spread of the COVID-19 outbreak, which can be used as a true description of the current condition of each country. For example, as shown in Figure 5, the low value of the parameter *γ* obtained directly from this model shows that the high *R*_0_ value, as in the case in Indonesia, is reinforced by the fact that cases and the spread of COVID-19 are still being experienced. On the other hand, as shown in Figure 2 and Figure 3, the high value of the parameter *γ* indicates that the *R*_0_ value is low, and that cases have been controlled or the process of spreading a COVID-19 outbreak is relatively small, as is the case in Singapore and Saudi Arabia.

It seems to be that the exception is the case of the Philippines, where the mean value for this parameter *γ* is in the range of some other countries discussed earlier, but is experienced with relatively mild effects. This can be assessed from the total number of people infected with COVID-19, which has started to decline. In this regard, this simple classic model of SIR can offer useful insights into not only the important *R*_0_-value, but also provide fast information on taking strict enforcement measures to prevent the severity of the COVID-19 epidemic.

Based on the estimation results of the parameters *β* (rate of infected cases) and *γ* (rate of recovered cases), epidemic cases in Singapore and Saudi Arabia have shown that these two parameters are close to the same value. This means that the process of infection and recovery have been a balance, with a decrease of the daily infected cases. Because these two parameters define the parameter *R*_0_, this parameter is also close to 1.00. This means that the impact of the virus transmission process has been well controlled. In the epidemic case of the Philippines, the two parameters did not approached each other significantly. While there is a decrease in daily infected cases, the prevention of the virus transmission process still needs to be improved. While the epidemic case in Indonesia is still ongoing, it can be seen from the results of the two estimation parameters, which do not have the same value, that the *β* parameter remains greater than the *γ* parameter, which shows that the impact of the epidemic severity is still large.

The dynamics of the COVID-19 pandemic in 35 European countries for 9 months have been predicted using a three-phase time series model. Multiple regression models were developed to predict the threshold of the COVID-19 incidence and the increase in mortality. The model was applied to analyze the association between deaths and COVID-19 incidents during the two waves that have occurred. The worse-affected and less-affected countries alternated between phases of the first and second waves. The conclusion drawn is that the incidence of COVID-19 and deaths are interrelated, and a link is seen during the two waves [35]. This interesting study showed that the classic SIR model can be applied to cases of the COVID-19 pandemic in 35 European countries for 9 months.

## Figures and Tables

**Table 1:** The SIR model parameters of various periods were obtained from data of cumulative infected cases in Singapore based on data compiled by WHO [34].

Period 2020	Parameter *β*	Parameter *γ*	Parameter *R*_0_	Value *R*^2^
23 January–23 May	0.135	0.121	1.115	0.94
23 January–23 June	0.134	0.124	1.080	0.95
23 January–23 September	0.130	0.127	1.024	0.96
23 January–23 December	0.126	0.125	1.008	0.97

**Table 2:** The SIR model parameters of various periods were obtained from data of cumulative infected cases in Saudi Arabia, based on data compiled by WHO [34].

Period 2020	Parameter *β*	Parameter *γ*	Parameter *R*_0_	Value *R*^2^
2 March–2 May	0.168	0.119	1.411	0.95
2 March–2 July	0.154	0.126	1.222	0.96
2 March–2 September	0.138	0.132	1.045	0.97
2 March–23 December	0.136	0.135	1.007	0.98

**Table 3:** The SIR model parameters of various periods were obtained from data of cumulative infected cases in the Philippines based on data compiled by WHO [34].

Period 2020	Parameter *β*	Parameter *γ*	Parameter *R*_0_	Value *R*^2^
23 January–23 May	0.153	0.118	1.296	0.95
23 January–23 June	0.150	0.124	1.209	0.97
23 January–23 September	0.145	0.128	1.132	0.98
23 January–23 December	0.141	0.130	1.084	0.98

**Table 4:** The SIR model parameters of various periods were obtained from data of cumulative infected cases in Indonesia based on data compiled by WHO [34].

Period 2020	Parameter *β*	Parameter *γ*	Parameter *R*_0_	Value *R*^2^
2 March–2 May	0.075	0.054	1.388	0.95
2 March–2 July	0.073	0.056	1.303	0.96
2 March–2 September	0.076	0.058	1.357	0.97
2 March–23 December	0.078	0.056	1.392	0.98

## Data Availability

Actual data comes from the World Health Organization (WHO) Coronavirus (COVID-19) dashboard which is available online: https://covid19.who.int// and accessed on 23 December 2020.
